# Beneficial Effects of Different Flavonoids on Vascular and Renal Function in L-NAME Hypertensive Rats

**DOI:** 10.3390/nu10040484

**Published:** 2018-04-13

**Authors:** M. Dolores Paredes, Paola Romecín, Noemí M. Atucha, Francisco O’Valle, Julián Castillo, M. Clara Ortiz, Joaquín García-Estañ

**Affiliations:** 1 Departamento de Fisiología, Facultad de Medicina & Instituto Murciano de Investigaciones Biosanitarias, Universidad de Murcia, 30120 Murcia, Spain; madopaca@um.es (M.D.P.); paodunromec@gmail.com (P.R.); ntma@um.es (N.M.A.); clara@um.es (M.C.O.); 2 Departamento de Anatomía Patológica, Facultad de Medicina, IBIMER, ibs.Granada, Universidad de Granada, 18011 Granada, Spain; fovalle@ugr.es; 3 Instituto Universitario de Envejecimiento & Research and Development Department, Nutrafur SA-FRUTAROM Group, 30820 Alcantarilla (Murcia), Spain; j.castillo@nutrafur.com

**Keywords:** flavonoids, nitric oxide, heart, kidney, sodium balance, phenylephrine, acetylcholine

## Abstract

Background: we have evaluated the antihypertensive effect of several flavonoid extracts in a rat model of arterial hypertension caused by chronic administration (6 weeks) of the nitric oxide synthesis inhibitor, L-NAME. Methods: Sprague Dawley rats received L-NAME alone or L-NAME plus flavonoid-rich vegetal extracts (Lemon, Grapefruit + Bitter Orange, and Cocoa) or purified flavonoids (Apigenin and Diosmin) for 6 weeks. Results: L-NAME treatment resulted in a marked elevation of blood pressure, and treatment with Apigenin, Lemon Extract, and Grapefruit + Bitter Orange extracts significantly reduced the elevated blood pressure of these animals. Apigenin and some of these flavonoids also ameliorated nitric oxide-dependent and -independent aortic vasodilation and elevated nitrite urinary excretion. End-organ abnormalities such as cardiac infarcts, hyaline arteriopathy and fibrinoid necrosis in coronary arteries and aorta were improved by these treatments, reducing the end-organ vascular damage. Conclusions: the flavonoids included in this study, specially apigenin, may be used as functional food ingredients with potential therapeutic benefit in arterial hypertension.

## 1. Introduction

Animal studies using flavonoid-rich foods are a valid alternative to advance the comprehension of the mechanisms underlying their hypotensive effects in experimental models of arterial hypertension [1]. The intake of polyphenols has been related with a beneficial effect that reduces the risk of hypertension [2]. In fact, epidemiological studies found that an increased consumption of foods and beverages rich in flavonoids is related to a reduced risk of cardiovascular death [3,4]. Moreover, the use of products with a natural origin that may cause few side-effects is an attractive possibility to be considered for the treatment of several pathologies [5]. Several studies have described that the consumption of flavonoid-rich food or isolated compounds improves several cardiovascular parameters such as flow-mediated dilation and cardiovascular risk biomarkers [6,7]. Additionally, many flavonoids induce the release of endothelium-derived vasodilatory factors such as nitric oxide (NO) or endothelium-derived hyperpolarizing factor (EDHF) and decrease the release of pro-inflammatory substances, thus, inducing an improvement of endothelial function [8,9].

L-NAME hypertension is a very frequently used model of endothelial dysfunction. Moreover, since L-NAME administration induces arterial hypertension, it has been used extensively to analyze the role of NO in the control of blood pressure [10,11,12]. The kidney seems to be one of the first organs that react to the loss of NO, and a reduced pressure natriuresis response and an enhanced role of the renin-angiotensin system have been implicated in its pathophysiology [10,11,12]. Arterial hypertension induced by chronic L-NAME administration is accompanied by cardiovascular remodeling, very evident in the heart and also in conduit and resistance vessels. Left ventricular hypertrophy and myocardial fibrosis [12,13] and thickening of the aortic wall and remodeling of mesenteric resistance arteries [14] have all been reported. Recently, down-regulated eNOS protein expression in blood vessels and depletion of plasma NO levels have been described in L-NAME-treated rats [15], thus probably contributing, by a reduced vasorelaxation, to increased vascular resistance and high blood pressure [16]. Increased levels of oxidative stress markers were also observed in L-NAME hypertensive rats [14], including peroxynitrite, a very reactive intermediate and one of the most potent oxidants known in biological systems, that causes long-lasting impairment of the vasoactive response to vasodilators [17]. Oxidative stress-derived products not only decrease NO bioavailability, causing impaired vasorelaxation, but also cause uncoupling of NOS to produce vasoconstrictor superoxide instead of vasodilator NO [17,18].

Therefore, the aim of the present study was to evaluate the vascular and renal effects of several flavonoid extracts in L-NAME-treated hypertensive rats. We have also examined some of the mechanisms involved in their beneficial effects such as an improvement in NO bioavailability and endothelial and vascular function, the reduction in oxidative stress markers and the effects on cardiovascular morphological changes.

## 2. Materials and Methods 

### 2.1. Animals

All the experiments were performed in male Sprague–Dawley rats (Harlan Lab., Barcelona, Spain) housed in a temperature-controlled environment, with 12:12-h light-dark cycle in the Animal Care Facility of the University of Murcia (REGAES300305440012). The animals were kept and treated according to the guidelines established by the European Union for the protection of animals used in experiments (86/609/EEC). All procedures were approved by the Animal Care and Use Committee of the University of Murcia (C1310050303). 

### 2.2. Experimental Groups

Eight- to nine-week-old rats, weighing 300–325 g, were randomized into seven groups: 1. Control (*n* = 6), rats without any treatment; 2. L-NAME (*n* = 6), rats receiving chronic L-NAME (*N*-w-nitro-l-arginine methyl ester, 10 mg/kg/day); 3. Apigenin (A, *n* = 6), rats simultaneously treated with L-NAME plus A (1.44 mg/kg/day); 4. Lemon Extract (LE, *n* = 6), rats simultaneously treated with L-NAME plus LE (2.84 mg/kg/day); 5. Grapefruit + Bitter Orange Extracts (GBO, *n* = 6), rats simultaneously treated with L-NAME plus GBO extract (9.28 mg/kg/day); 6. Cocoa Extract (COE, *n* = 6), rats simultaneously treated with L-NAME plus COE (2.52 mg/kg/day); 7. Diosmin (D, *n* = 6), rats simultaneously treated with L-NAME plus D (7.16 mg/kg/day).

A summary of the main features of the flavonoids used in the present study is available as a supplemental file. The extracts selected, were by virtue of their importance in the market, sales level, etc. and all of them were used as ingredients in nutritional supplements for many years. All of these extracts have been used with reasonable success in this market in the field of cardiovascular health, though perhaps non-specifically, given the diversity of their potential mechanisms of action and the corresponding physiological-macroscopic effects. The study used a single dose (mg/kg body weight/day) based on the usual market consumption, with a minimum adjustment to obtain dosages that supposed the same incidence in cost-dose/day (see Table S1 in Supplemental File). 

All treatments were administered over 6 weeks, in the drinking water, except for Diosmin that was given mixed with the powdered food, in powder feeders (Tecniplast, Radnor, PA, USA). All animals had free access to a standard rat diet with a 0.5% of sodium content (104 mEq/Kg) and tap water, with or without treatments. The concentrations of the drugs were adjusted daily according to the body weight and water and food intake. All products, except L-NAME (Sigma, St. Louis, MO, USA), were kindly provided by Nutrafur SA-FRUTAROM Group. 

The composition of the different extracts used in this study was determined by High-Performance Liquid Chromatography (HPLC) as previously described [19,20]. The HPLC chromatograms have been also included in the supplemental file. In all extracts and purified compounds assayed, the unique active components are flavonoids. A detailed and quantitative description is provided in Table S2. The rest of the components up to 100% of the extract composition were (depending on each extract): polysaccharides from the vegetable source used for the extraction (1–50%), water (3–5%), mineral salts (1–5%), pectins (1–5%) and lipids (1–2%). The molecular structures of main flavonoids are also described in Table S2.

### 2.3. Experimental Procedures

Rats were maintained in their cages up to weeks 4 and 5 when they were progressively accustomed to individual metabolic cages (Tecniplast, Radnor, PA, USA) three days a week. Then, the week 6th, after two days of adaptation, we measured food and water intake and urinary volume (diuresis) in 24 h. The urine samples were collected and centrifuged (1000× *g*, 10 min) to remove solid matter and then kept at −80 °C for further analysis. The urinary sodium concentration was determined using a sodium electrode (Thermo Scientific Orion, Waltham, MA, USA). Sodium balance (mEq/day/100 g) was calculated as the difference between sodium intake and urinary sodium excretion and factored by body weight. Sodium intake (mEq/day) was obtained by multiplying the consumption of food per day (g/day) by sodium content of the diet (0.104 mEq/g). Urinary sodium excretion (mEq/day) was determined as the product of sodium concentration and 24-h urinary volume (mL/day).

#### 2.3.1. Measurement of Blood Pressure and Samples Extraction 

After the metabolic study was completed, the animals were anesthetized with sodium pentobarbital (5 mg/Kg, i.p.) and placed on a heated table to maintain body temperature at 37 °C. A polyethylene catheter (PE-50) was placed in the right femoral artery to measure mean arterial pressure (MAP; Hewlett Packard 1280 pressure transducer and amplifier 8805D, Andover, MA, USA) and to collect blood samples, as previously described [10,11,12]. Then, blood was collected into heparinized tubes and plasma was obtained by centrifugation (1000× *g*, 10 min, 4 °C). Thereafter, the animal was euthanized by opening the thorax. We extracted the descending thoracic aorta and placed it in a Petri dish containing oxygenated and pre-warmed Krebs solution for the vascular reactivity study. Finally, kidneys, heart, and abdominal aorta were also removed. All samples were frozen (−80 °C) and a small portion was also fixed with a 10%-formalin solution for pathology studies. 

#### 2.3.2. Vascular Reactivity Study

The thoracic aorta was cleaned of adhering fat and connective tissue; care was taken not to disrupt vascular endothelium, as previously described [21]. Then, the aorta was cut into four rings (3–4 mm) and mounted in 10 mL organ baths (organ bath system LE 01004, Panlab, Barcelona, Spain) containing a physiological Krebs solution with the following composition (mM): NaCl, 118; KCl, 4.7; CaCl_2_, 2.5; MgSO_4_, 1.2; NaHCO_3_, 25; KH_2_PO_4_, 1.2; edetate calcium disodium, 0.026; and glucose, 5.6. The Krebs solution was maintained at 37 °C and continuously bubbled with a mixture of 95% O_2_ and 5% CO_2._ The rings are connected to isometric force transducers (TRI202P, Panlab) to detect tension changes that were acquired and analyzed with a data acquisition system (AD Instrument, Oxford, UK) consisting of a bridge amplifier (FE228), a data acquisition hardware (PowerLab 8/30) and a software (LabChart 6.0). Aortic rings were equilibrated for at least 45 min at a resting tension of 2 g before any specific experimental protocol was initiated. During this period, the bathing solution was replaced every 15 min and, if needed, the basal tone readjusted to 2 g. After the stabilization period, the aortic rings were constricted using a cumulative dose-response curve to phenylephrine (Phe, 10^−9^–10^−4^ mol/L), administered in 0.1 mL bolus. Then, the rings were washed (usually 2–3 times) until the resting tension was reached again and a second stabilization period of 30 min was allowed. To evaluate the vasodilator responses to acetylcholine (Ach), the aortic rings were pre-contracted with a submaximal dose of Phe (10^−6^ mol/L). Once a stable plateau was reached, a cumulative dose–response curve to the Ach (10^−9^–10^−4^ mol/L) was performed to assess the endothelium-dependent vasodilatation. Thereafter, the rings were frequently washed once again and a third stabilization period of 30 min was permitted and followed by an incubation period of 30 min with the NOS-inhibitor L-NAME (10^−4^ M) to inhibit NO synthesis. Next, a cumulative concentration-response curve to Ach was again performed, to evaluate the role of NO in the endothelium-dependent vasodilatation. Finally, we added a single dose of sodium nitroprusside (SNP, 10^−4^ M) to test the independent vasodilator responses and the functionality of the smooth muscle. The responses to Phe are expressed in grams and the relaxation to Ach and SNP as the percentage of the maximal Phe effect. Stock solutions of these drugs were prepared in distilled water and maintained frozen at −20 °C. Working solutions were prepared daily in Krebs solution. Drug concentrations are expressed as final bath concentrations. All reagents and vasoactive compounds were purchased from Sigma-Aldrich and Panreac (Barcelona, Spain). 

### 2.4. Analytical Procedures

TBARS (thiobarbituric acid reactive substances) in plasma and kidney tissue were determined as a measure of lipid peroxidation by using a colorimetric method [17]. Briefly, 0.5 mL of potassium phosphate buffer (0.1 M, pH 7.4) was added to 100 µL of plasma sample mixed or 50 μL of kidney tissue lysate. After mixing, 1 mL of reagent solution [1 mmol/L deferoxamine mesylate, 7.5% (w/v) trichloroacetic acid, 0.25 mol/L HCl and 0.37% thiobarbituric acid] was added and the mixture was vortex-mixed, covered with aluminium foil to avoid evaporation and heated at 90 °C for 15 min in a dry block heater (Heatblock II, VWR, Thorofare, NJ, USA). After the mixture had returned to room temperature, TBARS from standards (prepared from 1,1,3,3-tetraethoxypropane) and samples were extracted into 1 mL of butanol. After a vigorous vortex-mixing and a brief centrifugation (1000× *g* for 5 min), the absorbance of the butanol layer was read at 532 nm in a spectrophotometer (Eppendorf Biophotometer Plus, Hamburgo, Germany), and the value was expressed as nmol/mL of plasma or nmol/mg of kidney protein. The protein concentration was measured in the urine and lysates using the bicinchoninic acid based-method (Sigma). The plasma and urinary excretion of nitrite was determined by using the Griess reaction. Briefly, sample volumes of 100 µL were mixed with 50 µL of 1% Sulfanilamide in 5% Potassium Phosphate. Then 50 µL of 0.1% *N*-(1-Naphthyl) Ethyl-Enediamine dihydrochloride was added and incubated for 15 min. The nitrite concentration was quantified in a spectrophotometer at 540 nm against the standards and subtracting a blank from each individual sample. The final concentration was expressed in µg/mL for plasma or µg/day for urine samples.

### 2.5. Histopathological Analysis

Aortic, cardiac and renal tissue samples were fixed in 10% buffered formaldehyde and then processed, embedded in paraffin and sectioned (4 µm) as previously reported [22,23]. Transversal kidney, ventricular heart and thoracic and abdominal aorta sections were stained with hematoxylin-eosin and periodic acid-Schiff stain. The morphological study was done by a pathologist in blinded randomized sections of the tissues, with light microscopy and using the most appropriate stain for each lesion. The histo-morphometric measurements were performed with the software ImageJ 1.47 [24] (NIH, Bethesda, MD, USA). In the aorta, wall thickness was measured in three different, randomly selected regions, and also three times in each region. In the heart, different parameters of cardiovascular injury were analyzed in three transversal sections at different levels of the ventricle: (1) the inter-ventricular septum thickness was assessed in the middle central region of the cardiac cavities; (2) the number of all cardiac infarcts was counted in the three slides of each heart to evaluate the absence (0) or presence (1) of cardiac infarcts; (3) hyaline arteriopathy and (4) fibrinoid necrosis were also measured in a dichotomous manner depending on the absence (0) or presence (1) of these alterations; and (5) the relation between luminal diameter and wall thickness in main and intramural coronary arteries was obtained from five measurements of each artery. In the kidney, we evaluated the main alterations observed in transversal sections that included cortex and medulla. These were: (1) the absence or presence of hyaline arteriopathy in all the arteries seen in the whole section; (2) the relation between luminal diameter and wall thickness in the main renal artery or principal branches (if the first is missing); and (3) the absence (0) or presence (1) of tubular cast/cylinders in the cortical and medullary region from the entire kidney section. We did not observe any appreciable glomerular lesions and only other scarce vascular and tubular lesions. Finally, in order to estimate the overall vascular injury, we scored (0) if only one of the two organs, heart or kidney, was damaged and (1) if both organs were affected in the same animal.

### 2.6. Statistical Methods

Data are presented as the mean ± standard error. Differences between groups were compared mainly by one-way analysis of variance (ANOVA). In the vascular reactivity experiments, the values of EC_50_ were calculated from the individual curves and expressed as the negative logarithm (pEC_50_). Differences were considered statistically significant at a *p* level lower than 0.05.

## 3. Results

Body weight and hematocrit of all the experimental groups are listed in Table 1. After the six-week study period, L-NAME rats showed significantly lower body weight compared to control rats and all the flavonoid treatments showed a tendency to a normal body weight when compared to controls, especially in the case of apigenin. The hematocrit of all experimental groups was very similar, without significant differences between them.

### 3.1. Blood Pressure and Urinary Variables

**Figure 1:**
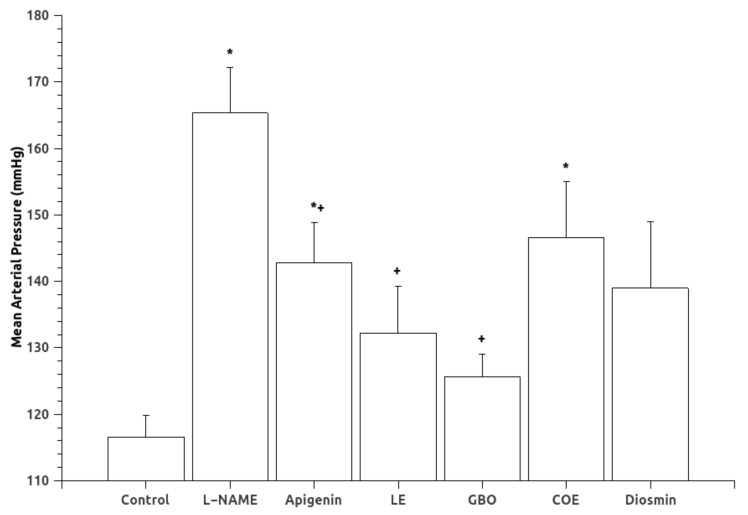
Mean arterial pressure (MAP) in the experimental groups. Abbreviations: L-NAME: (*N*(G)-Nitro-l-arginine methyl ester), LE (Lemon extract), GBO (Grapefruit + bitter orange extract), COE (Cocoa extract). Data are mean ± S.E.M. * *p* < 0.05 vs. Control; + *p* < 0.05 vs. L-NAME.

**Figure 2:**
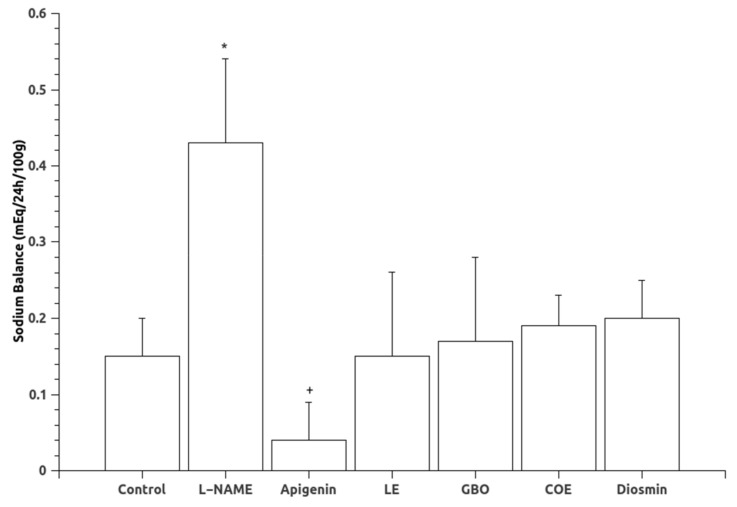
Sodium balance in the experimental groups. Abbreviations: L-NAME: (*N*(G)-Nitro-l-arginine methyl ester), LE (Lemon extract), GBO (Grapefruit + bitter orange extract), COE (Cocoa extract). Data are mean ± S.E.M. * *p* < 0.05 vs. Control; + *p* < 0.05 vs. L-NAME.

Mean arterial pressure (MAP) data are shown in Figure 1. Oral administration of L-NAME for six weeks caused a significant increase in mean arterial pressure. Treatments with A, LE and GBO extracts significantly reduced MAP associated with the chronic inhibition of NOS. Diuresis and natriuresis were not statistically different in the experimental groups (Table 1), although the L-NAME group had a tendency towards lower values. Regarding sodium balance, a greater sodium balance was found in the L-NAME treated group, indicative of sodium retention. The groups treated with flavonoids showed no statistical differences in sodium balance when compared to the control or L-NAME groups, although the group treated with apigenin showed a lower sodium balance than the L-NAME group (Figure 2).

**Figure 3:**
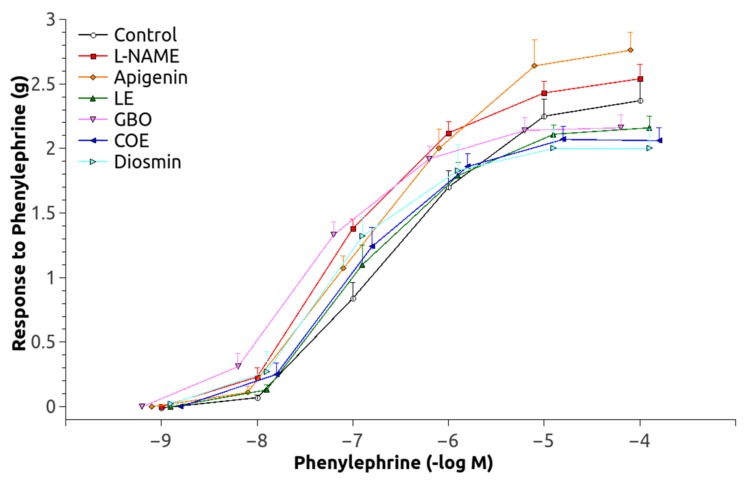
Pressor response to phenylephrine in aortic rings. Abbreviations: L-NAME: (*N*(G)-Nitro-l-arginine methyl ester), LE (Lemon extract), GBO (Grapefruit + bitter orange extract), COE (Cocoa extract).

**Figure 4:**
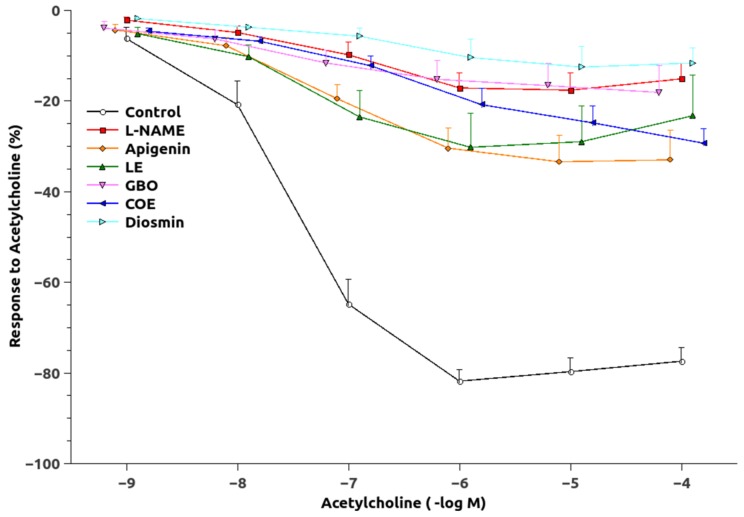
Vasodilatory response to acetylcholine in phenylephrine-preconstricted aortic rings. Abbreviations as in Figure 1.

**Figure 5:**
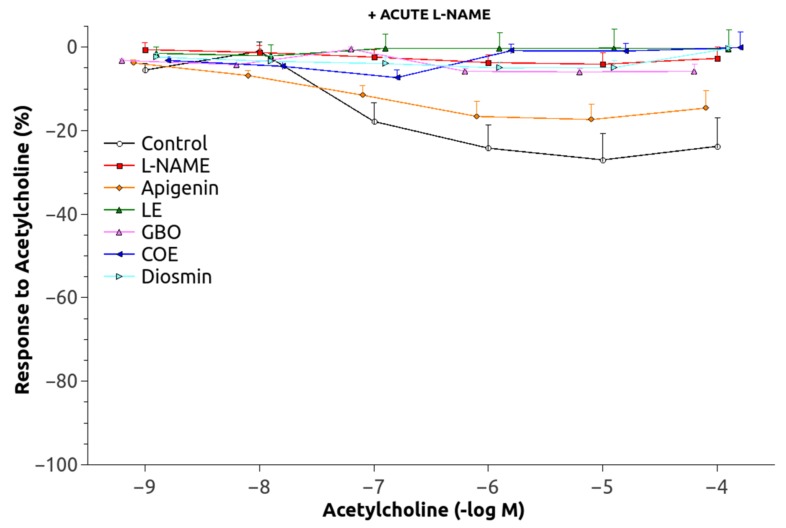
Vasodilatory response to acetylcholine in phenylephrine-preconstricted aortic rings, in the presence of acute L-NAME. Abbreviations as in Figure 1.

The dose-response curve to Phe was significantly shifted upwards in the animals chronically treated with L-NAME (Figure 3) and pEC_50_ values were significantly increased as compared to the controls (Table 2). The responses of the groups treated with L-NAME and flavonoids were not significantly different, but GBO, COE, and D also showed a greater pEC_50_ than that of the control group. Maximal Ach-induced vasodilatation (Figure 4) was significantly reduced in aortic rings from L-NAME hypertensive rats compared to control rats (Table 2). The relaxation to Ach improved in the aorta from rats treated with A, LE and COE but the relaxation still remained lower than in control rats. After administration of acute L-NAME (10^−4^ mol/L) to these aortic rings (Figure 5), the relaxant responses were further reduced, but there were some residual responses in the A and COE groups.

### 3.2. Vascular Function

Vasorelaxation in response to SNP was slightly but significantly lower in the flavonoid-untreated L-NAME rats when compared with the control rats. SNP induced similar responses in all flavonoid-treated groups, although A showed a significantly increased relaxation as compared to the untreated L-NAME group (Table 2).

### 3.3. Effect of Flavonoid Extracts on Oxidative Stress Status

Values of TBARS, nitrite and urinary protein excretion are shown in Table 3. Regarding TBARS, a significant increase was found only in the kidneys of the animals chronically treated with L-NAME as compared with controls. The rest of the groups did not show significant differences. Also, nitrite urinary excretion was significantly lower in the L-NAME group, but apigenin and diosmin significantly elevated it as compared to the L-NAME-treated group. There were no significant changes in proteinuria in the experimental groups, except in the L-NAME-treated group, showing enhanced urinary protein excretion, as compared with the control.

### 3.4. Histopathology Results

**Figure 6:**
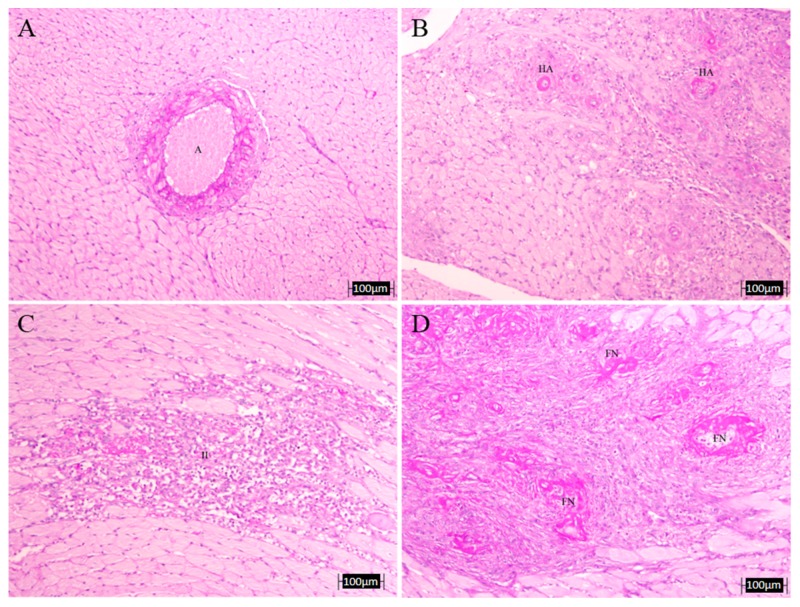
Representative microphotographs of heart lesions. Upper left: intramyocardial artery (**A**) of a control non-treated rat (no morphological alterations, PAS, 10×); (**B**) hyaline arteriopathy (HA) in a coronary artery of L-NAME-treated rat (PAS, 10×); (**C**) intense vascular damage with inflammatory infiltrate (II) and myocardiocytes lesions in a L-NAME-treated rat heart (PAS, 10×); (**D**) fibrinoid necrosis (FN) in a coronary artery of L-NAME-treated rat (PAS, 10×).

The analysis hearts (Table 4 and Figure 6) revealed that the L-NAME-treated group had more infarct zones, hyaline arteriopathy and fibrinoid necrosis as compared to the untreated control group. The treatments tend to decrease all these parameters. Wall–lumen ratio of coronary arteries was decreased in the L-NAME untreated rats when compared with control and, again, most treatments showed a tendency to increase the values but without being statistically significant. Interventricular heart septum thickness was significantly higher in the L-NAME-untreated animals compared to control rats and all treatments showed lower values, but only the decrease was significantly different in the groups treated with GBO and D. With respect to the thickness of the abdominal and thoracic aorta, L-NAME-treatment significantly increased it and LE and COE treatments reduced it to levels comparable to controls.

**Figure 7:**
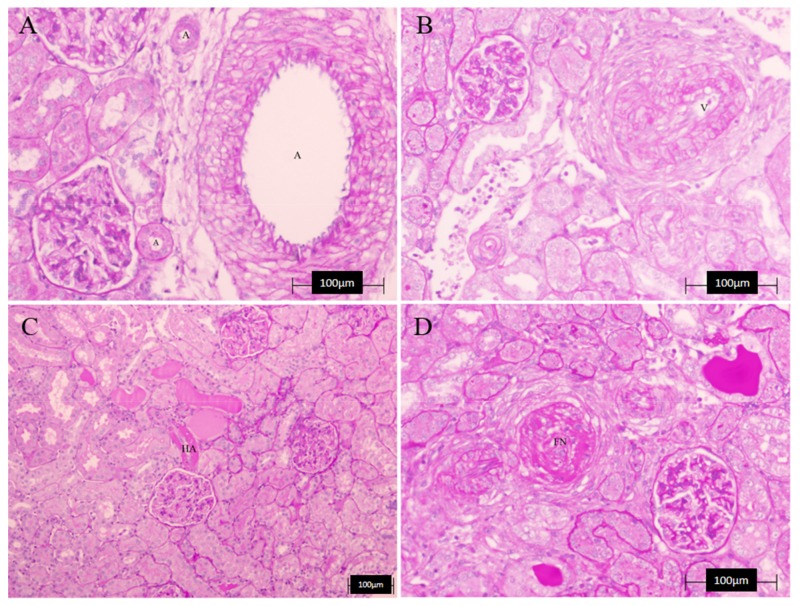
Representative microphotographs of kidney lesions. (**A**) renal artery (**A**) of a control non-treated rat (no morphological alterations, PAS, 20×); (**B**) muscular hypertrophy and lumen reduction in a renal artery (V) of L-NAME-treated rat (PAS, 20×); (**C**) hyaline arteriopathy (HA) and lumen reduction in a renal artery of L-NAME-treated rat (PAS, 10×); (**D**) fibrinoid necrosis (FN) in a renal artery of L-NAME-treated rat (PAS, 20×).

**Figure 8:**
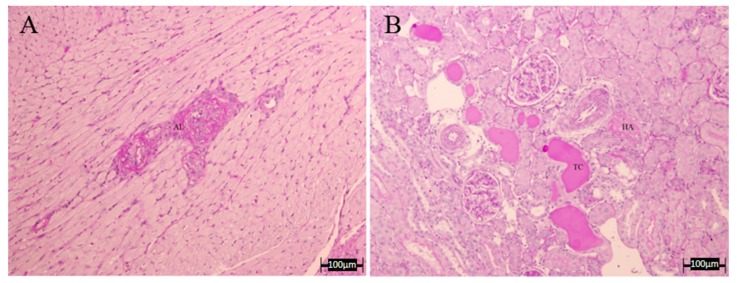
Representative microphotographs of heart and kidney lesion in the Apigenin group. (**A**) Less arterial and muscular injury (PAS 10×); (**B**) tubular cast and mild hyaline arteriopathy without fibrinoid necrosis in renal tissue (PAS, 10×).

Regarding the kidney (Table 5 and Figure 7), we found no significant differences between groups in any of the measured parameters, although for HA, all treated groups showed values lower than those of the L-NAME group, observing in the case of apigenin, a complete recovery. However, the presence of tubular cylinders was still evident in all the treated groups. Finally, when evaluating overall vascular damage (heart and kidney together), it seems that most treatments reduced it, with A, LE and COE showing lower vascular damage values, similar to the controls. Figure 8 shows a microphotograph representative of the beneficial effect of apigenin in those renal and cardiac lesions.

## 4. Discussion

The results of the present study show that some flavonoids, especially A, LE, and GBO, at the doses studied, reduced the elevated blood pressure levels reached by chronic L-NAME administration. This effect was accompanied, in the case of apigenin, with a normalization of the reduced vascular reactivity to vasoconstrictors and a lower sodium retention. An enhanced vasodilator ability, related to an increased production of NO, was also observed together with beneficial changes in the histopathological parameters in heart and kidney.

The chosen dose of each of the treatments responds to an objective criterion of a possible later use in humans. The doses ingested daily by the animals are very low compared to those used in other studies with similar compounds. Moreover, doses much greater than those applied in human therapy are usually used (5, 14, 18, 24). In addition, we needed doses that could be used with economic realism in the case of a future application to the field of pharmacy or the so-called nutritional supplements. In all cases, the doses used would cost 2–3 cents of euro per day, a value normally established as a reference in this type of products.

L-NAME-treated rats showed a lower weight than the controls and the treatment with flavonoids prevented the decrease in body weight during concomitant treatment with L-NAME (Table 1). This effect occurred despite a lower level of food intake in these flavonoid + L-NAME treated groups compared to L-NAME alone. This effect is probably related to the anti-hypertensive effect of these treatments, as other studies have shown with more specific treatments, such as blockade of the renin angiotensin system [12], a similar effect on body weight. However, other mechanisms such as the role of neuronal NOS in hypothalamus, the central regulator of food intake, may be involved. It is possible that flavonoids, by increasing neuronal NO bioavailability, may prevent the decrease in body weight. Thus, early studies showed that competitive inhibitors of NOS produced an l-arginine-reversible decrease in food intake [25], a result not found in the present studies, since the decrease in body weight was accompanied by a decrease in food intake. This contradiction could be explained by flavonoid effects on energy expenditure and digestive efficiency, as it has been recently demonstrated with naringenin [26].

Chronic NOS inhibition leads to sodium retention, also found in the present results, since NO is diuretic and natriuretic and promotes pressure natriuresis [10,11]. Although the treatments showed a tendency to improve sodium excretion (Figure 2), there were no significant differences between groups.

Many studies have reported a reduction in blood pressure following the consumption of flavonoid-rich products. In vitro studies have reported that flavonoids such as genistein, quercetin, and (−)-epicatechin regulated (directly or indirectly) NO production in isolated vessels or cultured endothelial cells [8,27,28]. However, most of them do not establish a clear dose-structure-activity relationship.

Our results agree with those studies showing that some of the treatments achieved a significant reduction of MAP, specifically GBO and LE and, at a lesser level, A and D (Figure 3). We hereby suggest that there are structural elements in the flavonoid molecular skeleton that are likely to achieve this BP lowering effect. The order of structural preference in the reduction of BP was similar when comparing the effectiveness of active flavonoids with that of a flavanone-glycoside predominance in the molecular structure, such as GBO and LE. A lower level would be that of flavone configurations, A and D, which are characterized by a double bond between carbons 2 and 3 conjugated with the *C*-4 carbonyl group (Table 1). It is also possible that the B-ring structure has some responsibility in this antihypertensive effect, since the structure 4′-hydroxy flavone/flavanone (A and naringin in GBO) seems to be more active than 3′-hydroxy-4′-methoxy flavone (D). The B-ring model 3′, 4′-dihydroxy (LE), thus, the catechol group is probably the most active structure at an equal concentration. Moreover, this structure seems to be essential for the inhibitory effect on angiotensin-converting enzyme activity, which plays a key role in the regulation of arterial blood pressure, independent of the presence of the flavone or flavanone flavonoid structure [20]. Future studies will be necessary to further define the structure-activity-dosage relationship of these drugs.

A mechanism responsible for the increase of BP during chronic L-NAME-treatment is associated with NO deficiency. Lower NO levels allow a greater expression of vasoconstrictors and attenuation of vasorelaxation in different vascular beds, as the present results confirm by the decrease in the EC_50_ of the L-NAME-treated group. As observed (Table 3), only apigenin (4′-hydroxy flavone) normalized the altered EC_50_ of these animals, although there was also some improvement in the LE group (3′, 4′-dihydroxy flavanone). This lower vasoconstrictor ability may be related to an increased production of some vasodilators, since the Ach-induced vasodilation was also improved by apigenin (Table 3, Figure 4 and Figure 5), especially in the group where NO was acutely inhibited. Interestingly, an NO-independent component seems to participate also in the vasodilatation improvement showed by A and COE since a residual vasorelaxation was still observed after acute L-NAME inhibition. This vascular relaxation promoted by flavonoids could partly explain the ability of these substances to reduce blood pressure. The results for A (4′-hydroxy flavone) and the significant lower efficacy of D (3′, 4′-methoxy flavone-7-*O*-glycoside), suggest that the combination of a double bond between carbons C2 = C3 (flavonoid-planar structure) as a glycon form (without sugar radicals) and with a B-ring type 4′-hydroxy could be of importance to produce vascular relaxation and the improvement of eNOS expression. Moreover, it appears that a small molecular volume is favorable for a given flavonoid to become active [29,30]. Our data are, thus, in agreement with previous findings from other studies [31,32,33].

Other mechanisms have been suggested to explain the increased endothelial NO bioavailability promoted by flavonoids. Several studies have shown that a regular consumption of flavonoids or flavonoid-rich foods can significantly improve the oxidative status as well as endothelial function [8]. To clarify and understand this, it is important to note that the antioxidant activity of flavonoids is not only related to a simple activity as oxygen free radical scavengers do. Other mechanisms that flavonoids use to regulate the oxidative status are related to an activity as epigenetic agents (increasing the expression of endogenous antioxidant enzymes as superoxide dismutase, glutathione) and/or as inhibitors of pro-oxidative enzymes (cyclooxygenase, lipoxygenase) from the arachidonic pathway [34,35,36,37]. In the present study, we detected a significant increase in ROS levels, as measured as TBARS (MDA: malonyl dialdehyde), in kidney tissue. It is important to note that MDA, an index of lipid peroxidation, has been found to be increased by L-NAME treatment [12,38,39]. It is likely that the reduction in kidney TBARS, observed in some of the flavonoid-treated groups (Table 4), is also contributing to the normalization of blood pressure. Although the results obtained do not show statistical significance, it may be interesting to consider that the treatments with a greater specific antioxidant efficacy are those having flavonoids with B-ring catechol structure (3′, 4′-dihydroxy), LE (eriocitrin) and COE (catechin compounds). The urinary nitrite excretion levels (Table 4) of the L-NAME-treated rats was lower than that of the control group. In the treatment groups, only A and D, thus, flavonoids with flavone structure, appear to generate a greater production of NO metabolites. Lower efficacy is shown by LE and COE, the catechol-containing flavonoid extracts.

It is known that the chronic deficit of NO usually results in an elevation of systemic blood pressure, an increase in glomerular capillary pressure and a reduction in the coefficient of ultrafiltration. These changes are associated with the presence of proteinuria and the development of glomerulosclerosis [40], as our data show (Table 4). The flavonoid treatments show, for most of them, a reduction in proteinuria, thus, indicating a reduction of renal glomerular damage. The behavior of A and LE is remarkable since they show values similar to those of the control group.

The metabolic and hemodynamic changes of L-NAME hypertension are also associated with the development of structural abnormalities, such as left ventricular hypertrophy, cardiac fibrosis, necrosis and protein remodeling, as well as with vascular wall hypertrophy [12], also shown in the present results. NO deficiency may, thus, result in increased monocyte and platelet adhesion, which by releasing growth factors would contribute to the thickening of the vascular wall. The proliferation was limited to the media, which is in agreement with the findings of others [41,42,43]. Although the lesions had a tendency to an improvement in the flavonoid-treated groups, no significant effects were seen in most of them. Only A, LE, and COE showed a beneficial effect in the aortic thickness. Regarding the renal structural changes, although we found no significant differences between groups, all treated groups showed values lower than the L-NAME group. Finally, when the overall vascular damage (heart and kidney together) was evaluated, it seems that most treatments reduced it, with A, LE, and COE showing lower vascular damage values, similar to the controls.

## 5. Conclusions

Our results suggest that the flavonoids included in this study, and already present in the market as nutritional supplements, may be used as food ingredients with potential therapeutic benefit in arterial hypertension. Further studies are necessary to elucidate the mechanisms involved in their antihypertensive effect, including an evaluation of the dose-activity relationship in order to determine the molecular structures most active. In any case, our results agree with previous findings [43] and suggest that the blood-pressure-lowering effect of these flavonoids may be related to a combination of vasodilator and antioxidant actions.

## Figures and Tables

**Table 1:** Body weight and hematocrit values in the experimental groups.

	Body Weight (g)	Hematocrit (%)	Food Intake (g/24 h)	Water Intake (mL/24 h)	Diuresis (mL/24 h)	Natriuresis (mEq/24 h)	Sodium Balance (mEq/24 h/100 g)
C	429.73 ± 6.36	47.38 ± 0.47	23.05 ± 0.68	35 ± 0.54	12.83 ± 1.46	0.41 ± 0.08	0.15 ± 0.05
L-N	345.73 ± 22.29 *	47.20 ± 1.20	21.1 ± 1.21	31.25 ± 4.7	7.85 ± 1.69	0.22 ± 0.06	0.43 ± 0.11 *
A	426.90 ± 11.46	50.67 ± 2.38	20.83 ± 0.63	30 ± 0.22	17.37 ± 3.85	0.47 ± 0.02	0.04 ± 0.05 †
LE	404.72 ± 16.96	49.83 ± 1.21	18.6 ± 0.49 *	25 ± 1.67 *	13.45 ± 2.79	0.33 ± 0.08	0.15 ± 0.11
GBO	392.65 ± 14.98	48.75 ± 3.35	16.1 ± 1.49 *	16.88 ± 2.48 *	10.65 ± 1.87	0.26 ± 0.08	0.17 ± 0.11
COE	399.38 ± 20.57	49.40 ± 1.47	16.58 ± 0.9 *	31.24 ± 5.6	21.84 ± 4.52	0.24 ± 0.06	0.19 ± 0.04

Abbreviations: C, control; L-N, *N*(G)-Nitro-l-arginine methyl ester treated group; A (Apigenin); LE (Lemon extract); GBO (Grapefruit + bitter orange extract); COE (Cocoa extract), and D (Diosmin). Data are mean ± S.E.M. * *p* < 0.05 vs. Control; † *p* < 0.05 vs. L-NAME.

**Table 2:** Contractile response to phenylephrine and maximal relaxation to acetylcholine and sodium nitroprussside in the experimental groups.

	Phenylephrine		Acetylcholine		SNP
Group	pEC_50_ (mol/L)	Maximal Contraction (g)	Maximal Relaxation (%)	After Acute L-NAME	Maximal Relaxation (%)
C	−6.45 ± 0.086	2.37 ± 0.15	82.70 ± 2.50	27.38 ± 6.54	99.38 ± 1.62
L-N	−6.83 ± 0.064 *	2.54 ± 0.12	19.88 ± 4.14 *	4.55 ± 2.70 *	89.71 ± 2.03 *
A	−6.56 ± 0.09 †	2.76 ± 0.37	35.87 ± 6.89 *	18.47 ± 3.92 †	96.14 ± 1.73
LE	−6.77 ± 0.12	2.16 ± 0.10	31.57 ± 8.23 *	5.74 ± 3.93 *	91.65 ± 3.12
GBO	−7.22 ± 0.18 *	2.18 ± 0.49	21.96 ± 5.32 *	7.67 ± 1.02 *	84.74 ± 2.93 *^,^†
COE	−7.08 ± 0.14 *	2.09 ± 0.02	29.63 ± 3.99 *	13.65 ± 2.40 †	90.50 ± 1.57 *
D	−7.00 ± 0.14 *	2.01 ± 0.34	13.78 ± 4.77 *	5.23 ± 1.81 *	91.59 ± 3.13

Data are mean ± S.E.M. Abbreviations as in Table 1. pEC_50_ is the negative logarithm of the half maximal effective concentration (EC50). * *p* < 0.05 vs. Control; † *p* < 0.05 vs. L-NAME.

**Table 3:** Measurements of TBARS, nitrite and proteinuria in the experimental groups.

	Plasma TBARS (nmol/mL)	Kidney TBARS (nmol/mg prot)	Urine TBARS (nmol/mg prot/24 h)	Plasma Nitrite (µg/mL)	Urinary Excretion of Nitrite (µg/24 h)	Urinary Protein Excretion (mg/24 h/kg bw)
C	1.5 ± 0.3	1.5 ± 0.1	576 ± 65	1.25 ± 0.18	11.83 ± 1.31	367.58 ± 11.19
L-N	1.8 ± 0.2	2.0 ± 0.3 *	437 ± 37	1.02 ± 0.05	6.39 ± 0.46 *	468.60 ± 39.52 *
A	1.7 ± 0.3	1.6 ± 0.2	512 ± 46	1.26 ± 0.09	13.40 ± 1.82 †	360.64 ± 40.15
LE	1.6 ± 0.3	1.1 ± 0.3	488 ± 69	0.88 ± 0.04	11.02 ± 1.63	373.50 ± 61.90
GBO	1.3 ± 0.2	1.6 ± 0.0	458 ± 51	1.02 ± 0.02	9.05 ± 1.64	426.51 ± 18.81 *
COE	1.3 ± 0.2	1.4 ± 0.3	482 ± 108	0.99 ± 0.11	14.12 ± 4.97	498.51 ± 67.17
D	1.4 ± 0.1	1.5 ± 0.1	546 ± 78	1.11 ± 0.12	12.02 ± 1.03 †	420.13 ± 75.24

Data are mean ± S.E.M. Abbreviations as in Table 1. * *p* < 0.05 vs. control, †, *p* < 0.05 vs. L-NAME.

**Table 4:** Histopathological results of the heart and aorta.

	# Infarcts	HA	FN	LWR	IVS	TAT	AOT
C	0.0	0.0	0.0	3.01 ± 0.58	2.41 ± 0.03	131.9 ± 3.1	110.7 ± 7.9
L-N	4.33 ± 3.84	0.67 ± 0.33	0.67 ± 0.33	2.22 ± 0.95	3.20 ± 0.10 *	176.3 ± 4.2 *	159.1 ± 6.5 *
A	0.67 ± 0.67	0.33 ± 0.33	0.0	2.39 ± 0.44	2.62 ± 0.25	178.0 ± 14.8 *	112.1 ± 5.9 †
LE	3.33 ± 3.33	0.0	0.0	2.97 ± 0.10	2.71 ± 0.16	141.4 ± 2.4 *^,^†	137.7 ± 3.7 *^,^†
GBO	3.67 ± 1.86	0.33 ± 0.33	0.33 ± 0.33	1.99 ± 0.40	2.42 ± 0.22 †	157.5 ± 8.5 *	144.5 ± 9.6 *
COE	2.67 ± 1.45	0.0	0.0	2.49 ± 0.06	3.11 ± 0.36	155.9 ± 5.4 *^,^†	135.2 ± 5.4 *^,^†
D	4.00 ± 4.00	0.33 ± 0.33	0.33 ± 0.33	2.74 ± 0.94	2.22 ± 0.08 †	164.3 ± 7.2 *	116 ± 4.4 †

Data are mean ± S.E.M. Abbreviations as in Table 2. HA, hyaline arteriopathy; FN, fibrinoid necrosis; LWR, lumen to wall ratio of coronary arteries; IVS, interventricular septum (µm); TAT, thoracic aorta wall thickness (µm); AOT, abdominal aorta wall thickness (µm); * *p* < 0.05 vs. control; † *p* < 0.05 vs. L-NAME.

**Table 5:** Histopathological results of the kidney.

	LWR	HA	TC	Combined Vascular Damage
C	1.80 ± 0.05	0	0	0
L-N	1.72 ± 0.32	0.67 ± 0.33	0.33 ± 0.33	0.67 ± 0.33
A	1.85 ± 0.27	0	0.33 ± 0.33	0
LE	1.07 ± 0.19	0.17 ± 0.17	0.50 ± 0.29	0
GBO	2.97 ± 1.07	0.33 ± 0.33	0.37 ± 0.33	0.33 ± 0.33
COE	1.63 ± 0.45	0.33 ± 0.33	1.00 ± 0.00	0
D	1.53 ± 0.09	0.33 ± 0.33	0.67 ± 0.33	0.33 ± 0.33

Data are mean ± S.E.M. Abbreviations as in Table 2. HA, hyaline arteriopathy; TC, tubular cilinders.

## Supplementary Materials

The following are available online at http://www.mdpi.com/2072-6643/10/4/484/s1, Table S1, Cost-dose per day of flavonoids used. Document: main features of flavonoids used and typical chromatograms of the products; Table S2: Characteristics of flavonoids used.
