# Supplementary material for: Beneficial Effects of Different Flavonoids on Vascular and Renal Function in L-NAME Hypertensive Rats
Source: Nutrients. 2018 Apr 13;10(4):484. doi: 10.3390/nu10040484 (PMC5946269; doi:10.3390/nu10040484)
Supplement: Supplementary file 1 [file nutrients-10-00484-s001.pdf]

Supplemental file Nutrients 284873, revision 1.

Table S1. Doses of flavonoids:

After several options and experiences, and considering that these were commercial ingredients, already existing as nutritional supplements, it was decided to establish a correlation between the usually recommended dose of intake and the objective of the same incidence in cost-dose / day. The following table summarize this:

Extract/compound	Recommended daily/dosage (mg)	Prize/kg (market) (euro)	Cost-dose/day (euro cents)	Animal dosage mg/kg/day (70 kg)
Diosmin (ref)	500	80-100	4-5	7.16
Apigenin	100	400-475	4-5	1.44
Lemon extract	200	230-260	4-5	2.84
Grapefruit + Bitter Orange extract	650	60-80	4-5	9.28
Cocoa extract	175	250-280	4-5	2.52

I. MAIN FEATURES OF FLAVONOIDS USED IN THE PRESENT STUDY.

1. **Lemon juice** has been commonly used by hypertensive patients in order to reduce acute blood pressure or as an alternative / complementary therapy on a chronic basis (Sari et al., 2012). Lemon (Citrus limon) contains significant concentrations of flavonoids in addition to known components such as carotenoids and ascorbic acid. The flavonoids present in lemon, are essentially glycosylated flavanones, especially of the rutinoside type and are eriocitrin (eriodictiol 7-O-β rutinoside) and hesperidin (Hesperetin 7-O-β rutinoside) (Miyake et al., 2007). Eriocitrin is a flavonoid with important antioxidant activity since it inhibits the autooxidation of lipids and has a suppressive effect on oxidative stress in diabetic rats (induced by streptozotocin and after acute exercise). Hesperidin has been shown to have anti-inflammatory and antihypertensive effects on experimental animals, although their antioxidant activity is lower in comparison with that of eriocitrin (Miyake et al., 2007). The product used in the study is a purified extract obtained from lemon peel.

2. **Grapefruit Extract + Bitter Orange:**

Grapefruit juice contains certain amounts of flavonoids mainly naringin (naringenin-7-O-neohesperidoside) and narirutine (naringenin-7-O-rutinoside) (Díaz-Juárez et al., 2009). Bitter orange contains important amounts of neohesperidine, a flavanone similar to hesperidin (found in sweet orange and lemon); it also contains naringin. Medical research has shown that grapefruit juice reduces the formation of atherosclerotic plaque and inhibits the cell proliferation of breast cancer and tumorigenesis of mammary cells. It has antioxidant, anti-nitrosamines, antiseptic, cardiotonic, hypocholesterolemic, and sedatives properties among several other functions (Kiani and Imam, 2007). In addition, in hypertensive subjects, the consumption of flavanones In grapefruit juice has a beneficial effect on blood pressure (Morand et al., 2011). In humans, Citrus Paradisi's

juice decreases also blood pressure, both systolic and diastolic in both normotensive and hypertensive patients (Díaz-Juárez et al., 2009). The product used in this study is an extract rich in glycosylated flavanones obtained from Immature grapefruit and bitter orange (1:1) fruits. Lemon, Grapefruit and Bitter Orange have been selected in this study because they are the most used sources in the preparation of nutritional supplements.

3. **Cocoa Extract :**

Cocoa flavonoids are flavan-3-ols or flavanols and include the monomeric forms, (-)-epicatechin and (+)-catechin, and the oligomeric form of the monomeric units, procyanidins (predominantly type B, which are epicatechin oligomers) (Fraga et al., 2011). These flavonoids are found in the seeds of the fruit of the cacao tree (Theobroma cacao) (Engler and Engler, 2004) and have been shown to improve endothelial function and decrease blood pressure in humans and animals (Fraga et al., 2011). The antihypertensive effect of products rich in cocoa has been related to:

1. The increase in plasma or urine of species derived from nitric oxide (NO).
2. The improvement of vasodilation due to NO.
3. The reduction of oxidative stress (Fraga et al., 2011).

Cocoa flavonoids favorably affect the lipoprotein profile. It has been described that the procyanidins of cocoa inhibit TNFα, a pro-inflammatory marker in the endothelial cells, thus reducing adhesion to the endothelium of T-lymphocytes (Quiñones et al., 2012). They also stimulate the formation of prostacyclin (PGI 2), an inhibitor of platelet aggregation, also inhibiting the formation of leukotrienes, vasoconstrictor agents and inflammation stimulants (Schramm et al., 2001). This extract was selected because it is probably the compound most studied at the cardiovascular level today and because2 it comes from the food with greater antioxidant capacity evaluated according to ORAC units.

4. **Apigenin:**

Apigenin is one of the most ubiquitous flavonoid compounds in the vegetal kingdom, being generally present in glycosylated form in one or more positions, being abundant in common fruits and vegetables such as parsley, celery, onions, chamomile and in some condiments (Patel et al., 2007). It has a variety of pharmacological activities including hypotensive, antiinflammatory, antispasmodic and antidiarrheal effects (Zhang et al., 2000), antioxidants, antimutagenic, antiproliferative, and anticarcinogenic effects (Patel et al. 2007). Apigenin has also been shown to abolish platelet responses induced by thromboxane A2 without substantially interfering with its synthesis (Guerrero et al. Al., 2005). In addition, it shows an interesting potential as a chemopreventive agent. In recent years, some of these anti-cancer mechanisms have been proposed including the modulation of the estrogenic/antiestrogenic activity, antiproliferative activity, induction of cell cycle and apoptosis, oxidation prevention, regulation of host system immune and

changes in cell signaling (Patel et al., 2007). The apigenin used in this study is a high purity molecule, not glycosylated and obtained through various stages of immature grapefruit fruits. It has been selected because it is a flavonoid with multiple demonstrated activities.

5. **Diosmin**:

Several pharmacological studies have shown that diosmin increases the venous tone, improves lymphatic drainage and protects the microcirculation. In edema, it produces a significant reduction in the circumference of the leg, thanks to its capacity to inhibit inflammatory reactions and to decrease capillary permeability. Regarding the skin, it has been shown to improve trophic venous disorders, such as stasis-induced and gravitational dermatitis and dermato fibrosclerosis (Ramelet, 2001). It has been selected because it is the flavonoid most used in pharmacology in diverse products aimed at the cardiovascular system.

II. HPLC Chromatograms of the flavonoids used in the present study.

A. Citrus related flavonoids: HPLC chromatograms using a C_{18} Lichrospher 100 analytical column (250 x 4 mm i.d.), with a particle size of 5 µm, termostated at 30˚C. The flow rate was 1 mL/min and the absorbance changes were monitored at 280 nm (Hewlett-Packard Series HP 1100 equipped with a diode array detector). Top to bottom: apigenin, diosmin, lemon extract and grapefruit+bitter orange extract, respectively (Benavente-García O, Castillo J, Lorente J, Ortuño A, Del Rio JA. Antioxidant activity of phenolics extracted from *Olea europaea* L. leaves. Food Chem 2000; 68: 457-462) (reference 19).

1. Apigenin.

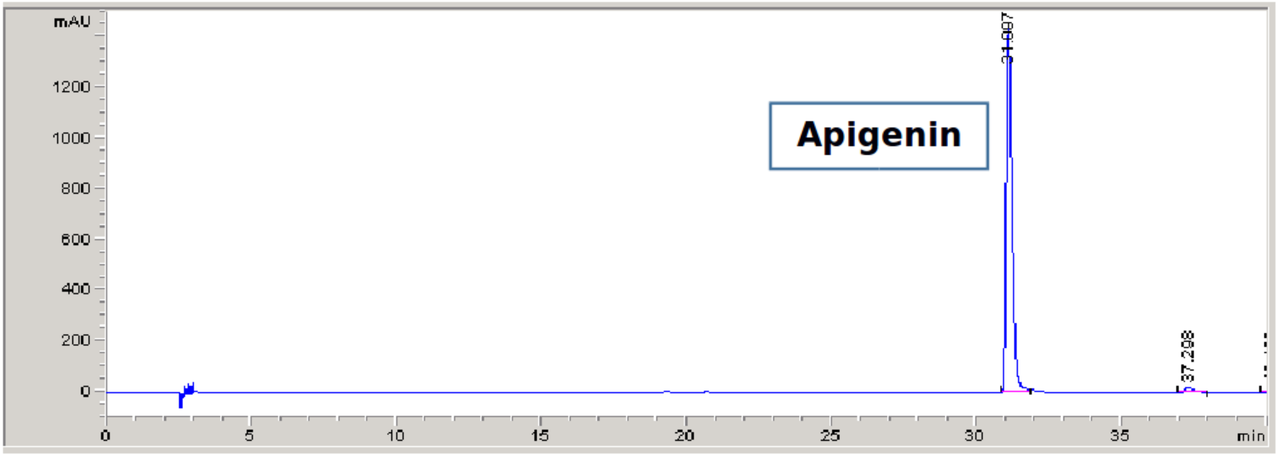

2. Diosmin

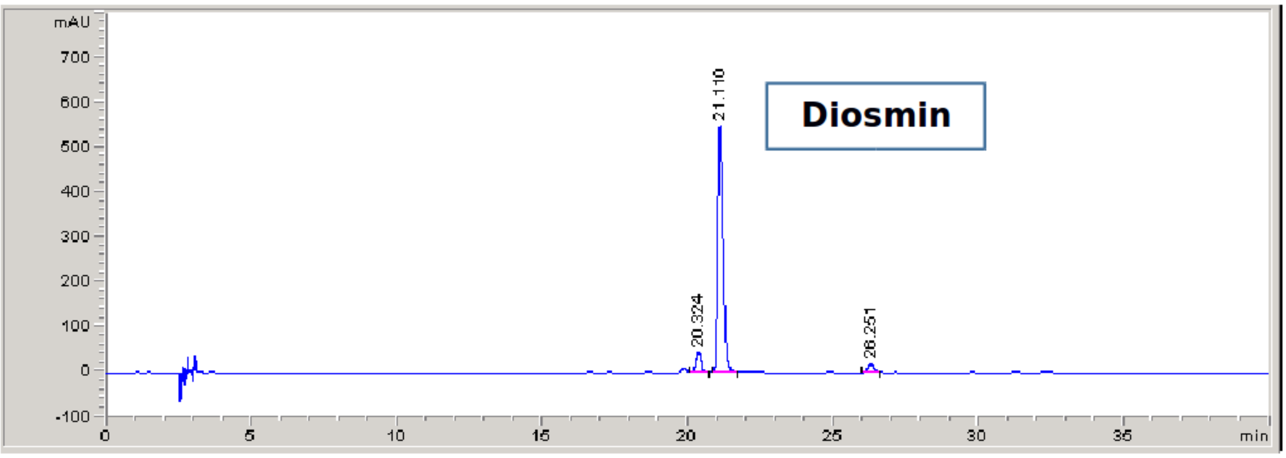

3. Eriocitrin

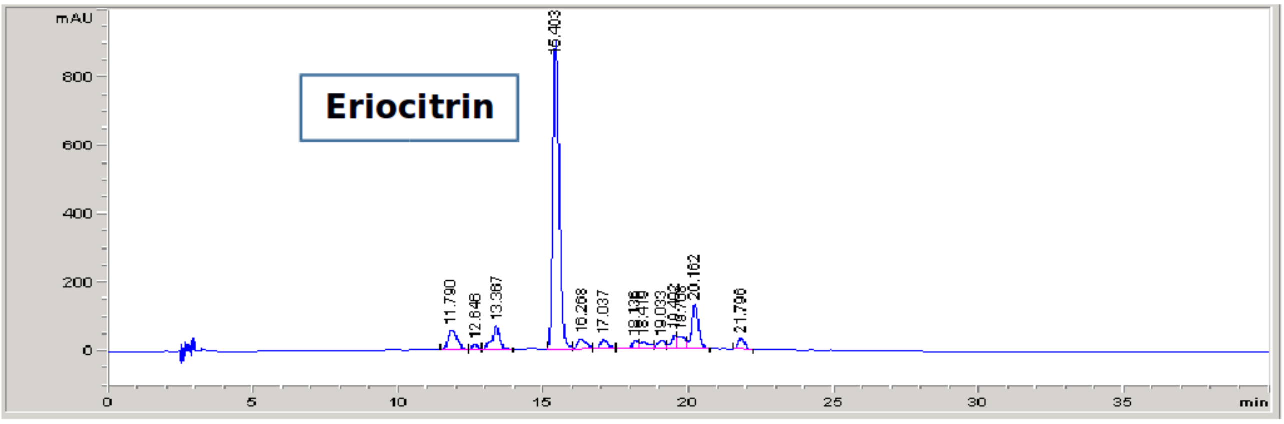

4. Naringin and Neohesperidin

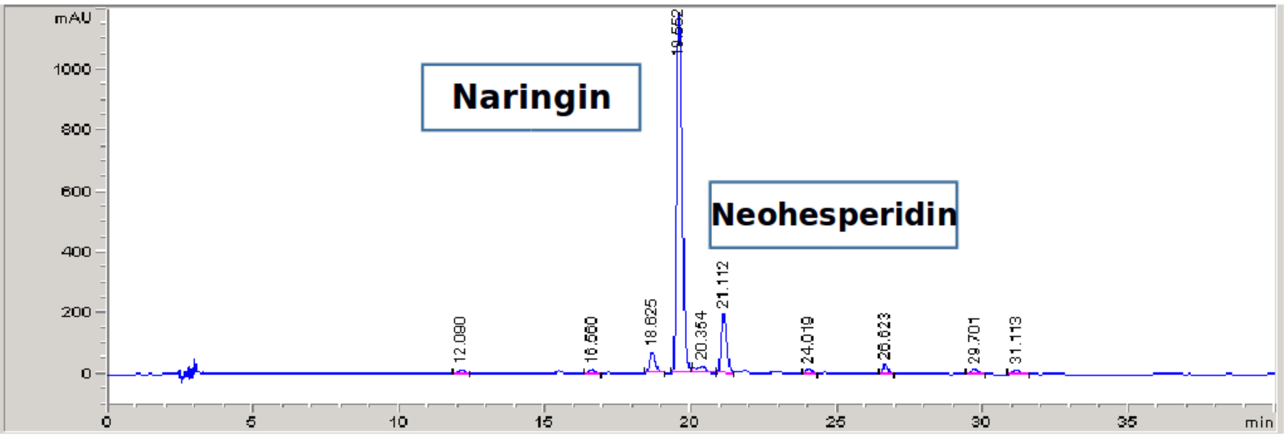

B. Cocoa extract: HPLC chromatograms using a C_{18} Lichrospher 100 analytical column (250 x 4 mm i.d.), with a particle size of 5 µm, termostated at 30˚C. The flow rate was 1 mL/min and the absorbance changes were monitored at 280 nm (Hitachi Elite Lachrom L-2130 equipped with a diode array detector). (Guerrero L, Castillo J, Quiñones M, Garcia-Vallvé S, Arola L, Pujadas G,

Muguerza B. Inhibition of angiotensin-converting enzyme activity by flavonoids: structure-activity relationship studies. PLoS One. 2012; 7: e49493) (reference 20).

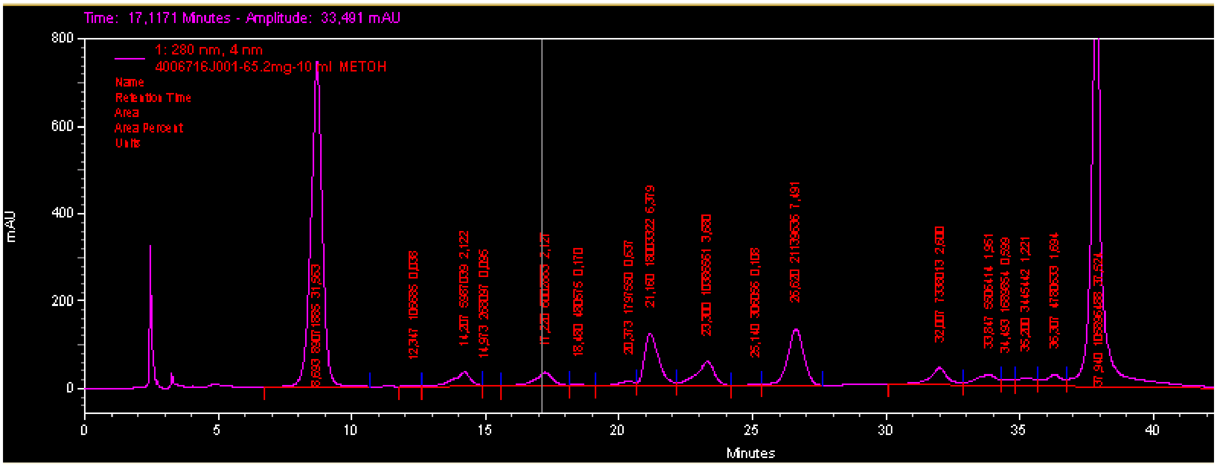

Table S2. Characteristics of flavonoids used.

Dosage (mg/day)	Chemical structure of major components (concentration % w/w)	Flavonoids skeleton / type of glycosidic structure	Main structural features
Flavonoid-rich vegetal extracts			
LE / (2,84)	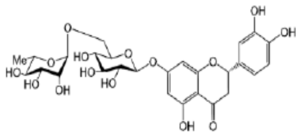 Eriocitrin (35%)	Flavanone-7-O-rhamnoglucoside type rutinoside	B ring with catechol group (3’, 4’-dihydroxy)
GBO / (9,28)	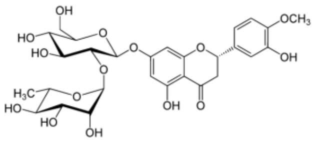 Neohesperidin (10.90%)	Flavanone-7-O-rhamnoglucoside type neohesperidoside	Neohesperidin: B ring 3’-hydroxy-4’-methoxy
	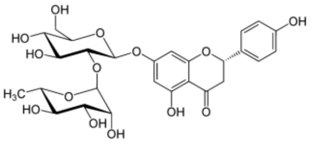 Naringin (54,30%)		Naringin: B Ring 4’-hydroxy
COE / (2,52)	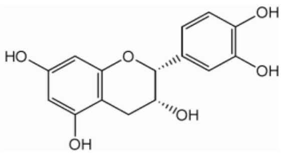 (-)-Epicatechin (6.84%)	Flavan-3-ol aglycone	B ring with catechol group (3’, 4’-dihydroxy) + C3-hydroxyl group

	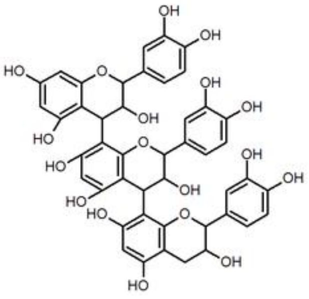<br>Procyanidin dimer B2 (4.88%)		
High purity flavonoids			
A / (1,44)	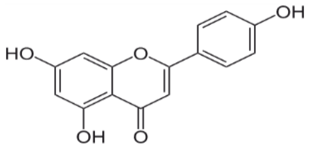<br>Apigenin (93.7%)	Flavone aglycone	B ring 4’-hydroxy + double bond C2=C3
D / (7,16)	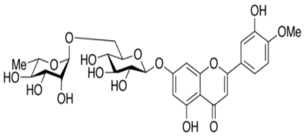<br>Diosmin (93.2%)	Flavone-7-O-rhamnoglucoside type rutinoside	B ring 3’-hydroxy-4’-methoxy + double bond C2=C3
